# Microbiome-Informed Precision Electroconvulsive Therapy: Oral–Gut–Immune Signatures and Seizure Biology as Candidate Predictors of Response—A Narrative Review

**DOI:** 10.3390/biomedicines14071467

**Published:** 2026-06-28

**Authors:** Bernard Rybczynski, Maciej Maslyk, Michal Pruc, Monika Janeczko, Iwona Niewiadomska, Lukasz Szarpak

**Affiliations:** 1 6th Department of Psychiatry, Mazovian Specialist Health Centre in Pruszkow, 05-802 Pruszkow, Poland; bernard.rybczynski@mscz.pl; 2 Institute of Molecular Biology, The John Paul II Catholic University of Lublin, 20-708 Lublin, Poland; maciej.malsyk@kul.pl (M.M.); monika.janeczko@kul.pl (M.J.); 3 Institute of Medical Science, The John Paul II Catholic University of Lublin, 20-708 Lublin, Poland; michal.pruc@kul.pl; 4 Institute of Psychology, The John Paul II Catholic University of Lublin, 20-950 Lublin, Poland; iwona.niewiadomska@kul.pl

**Keywords:** cognitive tolerability, electroconvulsive therapy, gut–brain axis, gut microbiota, inflammation, kynurenine, microbiome, oral microbiome, precision psychiatry, seizure duration

## Abstract

**Background/Objectives:** Electroconvulsive therapy (ECT) is among the most effective treatments for severe major depression, treatment-resistant depression, psychotic and bipolar depression, catatonia, and selected psychotic disorders. Yet response, remission, seizure adequacy, and cognitive tolerability remain difficult to predict for an individual patient. This review examines whether oral and gut microbial signatures can inform precision ECT as contextual biological markers, rather than as standalone explanations of ECT efficacy. **Methods:** A structured narrative PubMed/MEDLINE search was conducted on 1 May 2026 and supplemented by targeted manual searches of Crossref, Google Scholar, journal websites, and reference lists updated through 17 May 2026. Evidence was grouped as direct human ECT–microbiome studies, indirect human ECT biomarker studies, preclinical electroconvulsive shock (ECS) studies, and mechanistic microbiome–gut–brain literature. **Results:** Direct human ECT–microbiome evidence remains very limited and currently consists of two small prospective cohorts with sufficient microbiome data, totaling approximately 25 patients across studies, plus one single-patient case report. In severe or treatment-resistant depression, a pilot oral microbiome study with sufficient microbiological data from 14 patients reported higher pre-treatment oral alpha diversity in responders than in non-responders, without a consistent global oral microbiome shift after ECT. In schizophrenia, a small stool microbiome cohort of 11 patients suggested that baseline Bifidobacterium and Lactobacillus proportions may relate to symptom improvement, although sample size and confounding preclude firm inference. **Conclusions:** Microbiome-informed precision ECT remains a biologically plausible research direction, but current human evidence supports only cautious evaluation of baseline microbial context as a candidate predictor, not clinical microbiome-guided ECT, mediation, or microbiome-modifying intervention. The strongest current biological bridge comes from inflammatory markers, particularly baseline CRP and IL-6. Preclinical ECS studies support gut inflammatory, motility and vagal mechanisms, but they cannot substitute for human validation.

## 1. Introduction

Electroconvulsive therapy (ECT) occupies a singular position in contemporary psychiatry. It is simultaneously one of the most effective treatments for severe depressive illness and one of the most clinically misunderstood interventions in medicine [1,2]. In carefully selected patients with severe major depressive disorder, treatment-resistant depression, psychotic depression, bipolar depression, catatonia or selected psychotic disorders, ECT can produce a speed and magnitude of response that is rarely matched by pharmacotherapy alone [1,3]. Its underuse reflects not a lack of efficacy but the combined weight of stigma, limited service access, uncertainty about cognitive adverse effects and the persistent difficulty of predicting which patient will respond, how quickly, and at what tolerability cost [1,2].

The clinical challenge is familiar at the bedside. One patient with psychotic depression and psychomotor retardation may improve dramatically after several treatments; another, apparently similar in diagnosis and rating-scale severity, may require a prolonged course, experience short EEG seizures under propofol and benzodiazepine exposure, and show only partial improvement [1,4]. A catatonic patient may arrive dehydrated, immobile and constipated after weeks of reduced intake [1,3]. A patient with schizophrenia receiving clozapine may be referred for ECT with severe constipation, metabolic syndrome and anticholinergic burden [1,5]. These are not marginal details. They are the biological and clinical context in which ECT is delivered.

Conventional predictors of ECT response, including older age, psychotic features, psychomotor disturbance, shorter current episode duration, lower degree of antidepressant resistance and selected cognitive or illness-course variables, are useful at group level but remain insufficient for individual decision making [1,6,7]. Contemporary precision-ECT efforts have therefore moved toward integrative models combining clinical features with neuroimaging, electrophysiology, immune-inflammatory markers, epigenetic signals, molecular biomarkers and seizure parameters [8,9,10]. However, most models still omit an increasingly relevant biological system: the oral and gut microbiome.

The microbiome is not proposed here as an alternative explanation of how ECT works [11,12]. Such a claim would be premature and clinically unhelpful [13]. A more defensible question is whether pre-treatment oral and gut microbial states, microbial metabolites, gut barrier biology, inflammatory tone, autonomic regulation and gastrointestinal phenotype contribute to the biological context in which an induced therapeutic seizure is converted into neuroplastic and symptomatic responses [11,14,15,16,17]. In this formulation, microbiome-informed ECT is not about whether ECT merely changes bacteria; it is about whether microbial and immune–metabolic states help predict response, remission, seizure quality, cognitive tolerability, gastrointestinal symptoms and relapse.

This reframing is important because psychiatric microbiome research has often been centered on diagnosis. The literature has compared depression, bipolar disorder or schizophrenia with healthy controls, producing valuable but heterogeneous findings shaped by diet, smoking, medications, body mass index, constipation, hospitalization, sampling methods and analytic pipelines [18,19]. ECT provides a different clinical model: a time-limited biological intervention with measurable session-level physiology, standardized outcome definitions and repeated sampling opportunities [20]. The field is therefore well suited to ask whether microbial signatures add incremental value to precision psychiatry [21,22,23].

The direct human ECT–microbiome literature is small and should be interpreted accordingly. At present, it consists of two small prospective cohorts with sufficient microbiome data, totaling approximately 25 patients across studies, together with one single-patient case report. A prospective oral microbiome pilot study in severe or treatment-resistant depression reported that ECT responders had higher pre-treatment oral alpha diversity than non-responders, based on sufficient microbiological data from 14 patients, with no clear global oral microbiome shift after the ECT course [24]. A small prospective schizophrenia stool microbiome cohort including 11 patients found that baseline stool Bifidobacterium and Lactobacillus proportions were associated with symptomatic improvement after ECT, again without major pre–post microbial changes [25,26,27,28,29]. A single case report described a Clostridium change after ECT in schizophrenia, but this remains hypothesis-generating. These early observations suggest that baseline microbial ecology may be more relevant as a candidate predictor or contextual marker than as a simple downstream outcome [11,30].

This review synthesizes the evidence for microbiome-informed precision ECT. It deliberately separates three roles that are often conflated: microbiome as predictor, microbiome as mediator and microbiome as modifier. It also integrates seizure biology, inflammation, kynurenine metabolism, short-chain fatty acids, hypothalamic–pituitary–adrenal (HPA)-axis regulation, neuroplasticity, anesthetic exposure, cognitive tolerability, pharmacomicrobiomics, and gastrointestinal phenotype [11,31,32]. The aim is to provide a clinically disciplined framework for future studies rather than to overstate the readiness of microbiome testing for ECT practice.

## 2. Methods

This manuscript is a narrative review with a structured literature search and mechanistic synthesis. It was not designed as a systematic review or scoping review and therefore did not include protocol registration, duplicate independent screening, formal risk-of-bias assessment, quantitative synthesis, or PRISMA-ScR flow-diagram reporting. The structured search was used to improve transparency and reproducibility of the narrative evidence mapping, while the final synthesis remained interpretive and clinically oriented. Reporting was informed in spirit by PRISMA-ScR, a general transparency framework; no formal protocol, duplicate-screening workflow, quantitative synthesis, or PRISMA-ScR flow diagram was produced [33]. STORMS was not used as a reporting framework for the present review but was used as a reference point when formulating recommendations for future primary ECT–microbiome studies, particularly regarding sampling metadata, sequencing and bioinformatic workflow, contamination control, medication exposure, diet, gastrointestinal phenotype, oral-health metadata, and other clinically relevant confounders [34].

The core structured PubMed/MEDLINE search was conducted on 1 May 2026. Supplementary searches in Crossref, Google Scholar, journal websites, and reference lists were updated manually through 17 May 2026 to capture relevant articles published or indexed after the original draft, including 2025–2026 work on ECT biomarkers, microbiome pilot data, anesthetic effects, gastrointestinal motility, and inflammatory predictors. This manual update was narrative and targeted rather than a formal systematic-search update.

The core PubMed search string was: (“electroconvulsive therapy” OR “ECT” OR “electroconvulsive shock” OR “ECS”) AND (“microbiome” OR “microbiota” OR “dysbiosis” OR “gut microbiome” OR “gut microbiota” OR “oral microbiome” OR “oral microbiota” OR “salivary microbiome” OR “short-chain fatty acids” OR “SCFA” OR “butyrate” OR “propionate” OR “acetate” OR “kynurenine” OR “tryptophan” OR “lipopolysaccharide” OR “LPS” OR “zonulin” OR “intestinal permeability” OR “gut-brain axis” OR “microbiota-gut-brain axis” OR “adenosine” OR “purine” OR “bile acids” OR “indole”) AND (“response” OR “remission” OR “predictor” OR “biomarker” OR “treatment outcome” OR “seizure duration” OR “seizure quality” OR “cognition” OR “memory” OR “constipation” OR “gastrointestinal symptoms”).

Eligible evidence included human ECT studies with oral microbiome, gut microbiome, stool microbiota, salivary microbiome or microbial metabolite measures; human ECT studies with inflammatory, kynurenine, metabolomic or gut–brain-relevant biomarkers; human ECT studies on seizure duration, seizure quality, seizure threshold, medication effects, anesthetics or cognitive tolerability; studies on gastrointestinal symptoms, constipation, autonomic changes or gut motility during ECT; and ECS animal studies examining microbiome composition, gut inflammation, gut motility, vagal signaling, microbial metabolites or depressive-like behavior. Broader microbiome–gut–brain literature was included only when it directly supported an ECT-specific mechanism. The evidence domains, search focus, interpretive role and methodological safeguards used for evidence classification are summarized in Table 1.

Exclusion criteria were general depression–microbiome or schizophrenia–microbiome studies without ECT relevance, probiotic trials without ECT relevance, neuromodulation studies involving rTMS, tDCS, DBS, VNS or electroacupuncture unless used only as contextual evidence, opinion pieces without primary data, and non-peer-reviewed preprints unless explicitly labeled as hypothesis-generating. The final narrative corpus discussed in detail comprised direct human ECT–microbiome studies, ECT biomarker and seizure-parameter studies, ECS translational studies, microbiome methodology papers, and mechanistic microbiome–gut–brain studies relevant to inflammation, HPA-axis tone, kynurenine biology, SCFAs, gut barrier function, oral microbiome ecology and pharmacomicrobiomics. Key human and translational studies informing the synthesis are summarized in Supplementary Table S1.

Evidence was interpreted with five safeguards. First, oral and gut microbiomes were treated as distinct compartments rather than interchangeable measures. Second, predictor, mediator and modifier hypotheses were separated. Third, direct human ECT evidence was distinguished from indirect biomarker evidence and from animal ECS data. Fourth, microbial taxa were not overinterpreted without considering function, metabolites and compositional data constraints. Fifth, medication, diet, body mass index, smoking, antibiotics, proton-pump inhibitors, laxatives, constipation, oral hygiene, periodontal disease, hospitalization and session-level ECT protocol variables were treated as central confounders. The rationale for this conservative approach is reinforced by the large inter-individual variation in the healthy human microbiome and population-level microbiome structure [35,36].

## 3. Current Predictors of ECT Response: Why New Biomarkers Are Needed

ECT response is clinically patterned but individually uncertain. Meta-analytic evidence supports older age, psychotic features, psychomotor disturbance, shorter episode duration and lower treatment resistance as predictors of better response or remission in major depression [6,37,38]. These variables remain important because they represent readily available clinical knowledge. A severely melancholic or psychotic depression with prominent psychomotor slowing is often a different ECT proposition from a long-standing nonpsychotic depression complicated by personality pathology, benzodiazepine dependence, chronic pain, metabolic disease and polypharmacy [1,39].

The problem is that these predictors do not provide enough precision at the individual level. The Dutch ECT Consortium prediction model illustrates both progress and limitation: readily available clinical variables can reduce uncertainty, but substantial variability remains unexplained [40]. Recent narrative and systematic work on ECT biomarkers similarly emphasize that immune-inflammatory markers, neuroimaging, epigenetic markers, electrophysiology and clinical symptoms converge only partially and remain difficult to implement at the bedside [7,20,41]. Precision ECT therefore requires additive, biologically coherent variables, not isolated new biomarkers [42].

Seizure physiology is fundamental to this problem. ECT is not simply a procedure that delivers electricity; it is a treatment that induces a controlled generalized seizure under anesthesia. Clinical outcome depends on electrode placement, pulse width, stimulus dosing relative to seizure threshold, EEG seizure duration, postictal suppression, number of treatments, anesthetic agent and concomitant medications [1,43,44]. A large Swedish registry study of 6998 patients with major depressive disorder treated with unilateral ECT found that EEG seizure duration was associated with remission and that anticonvulsants and benzodiazepines were associated with shorter seizures and less favorable outcomes [42]. This does not make seizure duration a perfect surrogate for treatment quality, but it confirms that any biological predictor must be interpreted in relation to session-level ECT physiology [42,45].

Inflammation is currently a more mature bridge than the microbiome itself. A 2025 meta-analysis of 14 studies including 556 patients with depression reported that higher baseline CRP and IL-6 were associated with greater depressive symptom reduction after ECT [46]. The same literature also suggested a role for kynurenine metabolites and IL-8 dynamics [46,47]. These findings fit a broader model in which inflammatory depression may be more responsive to ECT-induced neuroimmune and neuroplastic modulation, while also providing a rational entry point for microbiome biology [20,41,48].

Microbiome-informed ECT should therefore be evaluated not as a competing predictor but as a potential layer in a multimodal model. The clinically meaningful question is whether oral or gut microbial data improve prediction beyond age, psychosis, episode duration, treatment resistance, baseline cognition, CRP/IL-6, medication exposure and seizure quality [41,49,50,51]. This incremental-value criterion should define the field.

## 4. Oral Microbiome and ECT Response in Severe Depression

Within this very small direct evidence base, the most directly relevant human depression evidence comes from the prospective oral microbiome pilot study by Ammer-Herrmenau and colleagues [24]. The investigators enrolled patients with severe or treatment-resistant depression undergoing ECT and collected oral swabs before and after treatment. Sufficient microbiological data were available for 14 patients, divided into seven responders and seven non-responders using the conventional criterion of at least 50% reduction in Montgomery–Åsberg Depression Rating Scale score [24,49]. The small sample size precludes definitive inference, but the study is important because it places the oral microbiome inside the ECT prediction problem rather than inside a generic depression–microbiome framework [11,24,51]. The study is summarized in Supplementary Table S1, Ammer-Herrmenau et al. [24] row.

The central observation was that responders had higher pre-treatment oral alpha diversity than non-responders [24,52]. Importantly, the study did not show a robust global oral microbiome shift after the ECT course. Responders and non-responders reportedly did not differ substantially in baseline depression severity or in key ECT treatment variables, although age and atypical antipsychotic exposure remained clinically relevant factors [53]. This pattern favors a baseline-predictor interpretation: oral microbial ecology may reflect a biological state that makes response more likely, rather than a microbial change caused by ECT [11,16,52].

The oral compartment deserves attention for pragmatic and biological reasons. It is accessible, acceptable to patients, feasible for repeated sampling during an ECT course and potentially easier to integrate into clinical workflows than stool collection [54,55]. It may also reflect systemic inflammatory state, salivary immune activity, medication exposure, xerostomia, smoking, diet, dental disease and oral hygiene [56]. Recent population-based and review work increasingly supports the oral–brain axis as an underexplored component of mental health research [17,51]. However, the oral microbiome should not be treated as a proxy for stool microbiota. It is its own ecological niche, with distinct local determinants and distinct sources of confounding [57,58,59].

Taxonomic signals from the pilot study should be handled with restraint. Streptococcus-dominant patterns were described more often among non-responders, while Gemella and Aggregatibacter signals were noted among responders. These observations are not clinical biomarkers. Streptococcus is a dominant oral genus and may reflect plaque biofilm ecology, salivary flow, diet, xerostomia, dental disease, antibiotic history, smoking, hospitalization or local mucosal inflammation rather than an ECT-specific biological state [22,23]. Gemella is also a common oral and upper aerodigestive commensal, and its clinical meaning depends strongly on species-level identity, co-occurring taxa and host context. Aggregatibacter includes species linked to periodontal inflammation, particularly A. actinomycetemcomitans, but genus-level detection alone cannot determine whether the signal reflects periodontal disease, oral dysbiosis or systemic immune relevance [59,60]. Therefore, these taxa should be interpreted as markers of oral ecological context, not as functional predictors of ECT response. For a top-tier ECT–microbiome study, species names are less important than reproducible functional signatures, inflammatory markers and metabolite profiles [16,52].

A clinically experienced interpretation is that oral microbiome sampling may become useful only if embedded within oral-health, medication, inflammatory and ECT-procedural metadata, rather than interpreted as an isolated microbial readout [16,55,61].

## 5. Gut Microbiome and ECT Response in Schizophrenia and Psychotic Disorders

Direct stool microbiome evidence in ECT-treated psychotic disorders is very limited. Kanayama and colleagues studied 11 patients with schizophrenia receiving ECT and examined stool bacterial proportions before and after treatment [62]. In the primary linear regression model for change in BPRS score, baseline Bifidobacterium abundance showed a positive association with symptom improvement (β = 2.383, *p* = 0.023), whereas baseline Lactobacillus abundance showed an inverse association (β = −1.903, *p* = 0.020). For catatonia severity, the corresponding BFCRS associations did not reach statistical significance (Bifidobacterium: β = 2.314, *p* = 0.093; Lactobacillus: β = −1.416, *p* = 0.132). Confidence intervals were not reported, and the sample size was only 11 patients; these coefficients should therefore be interpreted as exploratory regression signals rather than stable effect estimates. No bacterial group showed a significant pre–post change in proportion. Like the depression oral microbiome pilot, this finding supports a baseline-predictor hypothesis more than a simple post-ECT-change hypothesis. This cohort is summarized in Supplementary Table S1, Kanayama et al. [63] row.

The quantitative interpretability of this schizophrenia cohort is limited. The study reported directional associations between baseline bacterial proportions and symptomatic improvement, but the sample size was too small to derive clinically meaningful thresholds or stable multivariable estimates. Effect sizes and confidence intervals, where unavailable or insufficiently precise, should therefore not be inferred from the reported associations. The Bifidobacterium and Lactobacillus findings should be presented as exploratory signals requiring replication rather than as validated predictors of ECT response.

The clinical context of schizophrenia makes this signal interesting but fragile. Patients referred for ECT in schizophrenia are often complex: treatment-resistant psychosis, catatonia, severe agitation, clozapine augmentation, polypharmacy, metabolic syndrome, smoking, low physical activity, constipation and variable inpatient diet are all common [63,64,65]. Antipsychotic exposure is not merely a medication variable; it can change weight, insulin resistance, appetite, bowel transit and microbial ecology. Clozapine and olanzapine are particularly important because they are associated with metabolic changes and gastrointestinal hypomotility that can reshape stool microbiota and microbial metabolites [63,66,67,68,69].

The 2019 case report describing decreased Clostridium abundance after ECT in schizophrenia should be cited only as hypothesis-generating [62]. This case report is summarized in Supplementary Table S1, Kanayama et al. [70] row. Single-case microbial trajectories are vulnerable to diet, antibiotics, laxatives, sampling timing, bowel transit and analytic noise [71,72]. Their value is not to establish causality but to show why repeated sampling and careful metadata are needed. In ECT-treated psychosis cohorts, constipation and clozapine exposure should be treated as core biological variables, not as footnotes [64].

Broader schizophrenia microbiome studies provide mechanistic plausibility but not ECT evidence. Fecal microbiota transfer studies have linked schizophrenia-associated microbiota to behavioral changes, glutamate–glutamine–GABA cycle disturbances and kynurenine pathway abnormalities in mice [68,69]. These findings align with ECT-relevant pathways, but they do not show that gut microbiota predicts ECT response. For this reason, they should be used as mechanistic background, not as evidence for clinical microbial stratification [29,53,71].

In psychotic disorders, the most useful future ECT–microbiome cohorts will likely be diagnosis-specific or stratified by clinical indication. Catatonia, clozapine-resistant schizophrenia and acute psychotic agitation are biologically and clinically different reasons for ECT referral [1,73,74]. Combining them without phenotypic clarity would obscure signals. A pragmatic strategy would be to treat schizophrenia and catatonia as exploratory cohorts while focusing primary statistical power on severe depressive illness, where ECT outcome definitions and comparative data are stronger.

## 6. Preclinical ECS Evidence: Microbiota, Gut Inflammation, Vagal Signaling and Depressive-like Behavior

Electroconvulsive shock (ECS) animal models are not clinical ECT, but they allow experimental interrogation of pathways that are difficult to study in acutely ill patients. Their value is mechanistic: investigators can control stress exposure, collect gut tissue, measure local cytokines, test motility, manipulate the vagus nerve and examine depressive-like behaviors [75,76,77]. Their limitation is equally clear: mouse taxa, stress models and ECS protocols do not translate directly into human ECT biomarkers.

In a chronic unpredictable mild stress model, Ji and colleagues reported that ECS/ECT-like treatment improved depressive-like behavior, reduced gut inflammatory cytokines including TNF-alpha, IL-6 and IL-1beta, and altered microbial diversity and taxa [11]. The finding supports a gut inflammatory pathway but does not justify species-level extrapolation. In translational terms, the important message is that seizure-based neuromodulation may interact with intestinal inflammation and microbial ecology in a bidirectional manner [11,78,79]. This preclinical evidence is summarized in Supplementary Table S1, Ji et al. [80] row.

Dai and colleagues provided a complementary gut physiology signal [81]. In a clinical component, ECT was associated with improvement in gastrointestinal symptoms, particularly constipation, among patients with major depressive disorder [81]. In a depressive-like mouse model, ECS improved distal colonic motility, and subdiaphragmatic vagotomy attenuated this effect. This supports involvement of a hypothalamic paraventricular nucleus–vagus–gut pathway [79,81]. For microbiome-informed ECT, this is highly relevant because gut transit time is a major determinant of stool microbial composition and metabolite concentration [82,83,84]. The clinical and translational findings are summarized in Supplementary Table S1, Dai et al. [81] row.

Because ECS studies are heterogeneous and cannot be read as direct human ECT evidence, Table 2 summarizes the main preclinical signals and their translational boundaries. The purpose of the table is not to upgrade ECS findings to clinical biomarkers but to specify which findings are mechanistically useful and which should not be directly extrapolated to patient-level ECT prediction or treatment selection.

These studies sharpen a methodological point. If ECT changes stool microbiota after treatment, the change could reflect improved mood, improved appetite, hospital diet, increased mobility, altered laxative use, decreased constipation, medication changes, autonomic shifts or a direct gut–brain effect of the induced seizure. A pre–post microbial change is therefore not automatically a mechanism of antidepressant response. It may be a secondary marker of altered gut physiology [62,83,85,86].

**Table 2:** Preclinical ECS findings and limits of clinical translation.

ECS Finding Domain	Main Preclinical Observation	Mechanistic Value	Not Directly Clinically Translatable
Gut inflammatory signaling	ECS-like treatment has been associated with reduced gut inflammatory cytokines, including TNF-alpha, IL-6 and IL-1beta, in stress-based animal models [11,77].	Supports a plausible gut–immune pathway linking seizure-based neuromodulation with intestinal inflammatory tone.	Does not prove that human ECT improves depression through gut cytokine changes or that gut cytokines can guide ECT decisions.
Gut motility and vagal signaling	ECS improved distal colonic motility in depressive-like mice, and this effect was attenuated by subdiaphragmatic vagotomy [79].	Supports involvement of vagal/autonomic gut–brain pathways and highlights transit time as a major microbiome confounder.	Does not establish that human ECT-induced microbiome changes are causal or that motility effects predict psychiatric response.
Microbial diversity and taxonomic shifts	Animal ECS studies have reported changes in microbial diversity and taxa after ECS-like treatment [11,77].	Generates hypotheses about host physiology, stress, diet, gut inflammation and microbial ecology after seizure-based intervention.	Specific microbial taxa identified in animals should not be interpreted as human ECT biomarkers because of species differences, model constraints and protocol heterogeneity.
Depressive-like behavior	ECS-like treatment improved depressive-like behaviors in selected animal models [11,77].	Provides experimental support for studying gut–brain mechanisms alongside seizure-based neuromodulation.	Behavioral improvement in rodent stress models cannot be directly mapped onto remission, cognitive tolerability or relapse after clinical ECT.
Purinergic or microbial metabolite hypotheses	Preclinical and mechanistic literature suggests possible links between microbial metabolites, purinergic signaling, adenosine biology and seizure physiology [86,87].	May inform future metabolomic panels and mechanistic experiments.	These signals remain hypothesis-generating and do not justify clinical microbiome-guided ECT or microbiome-modifying interventions.

Abbreviations: ECS, electroconvulsive shock; ECT, electroconvulsive therapy; IL, interleukin; TNF-alpha, tumor necrosis factor-alpha.

Preclinical reports involving microbiota-derived purines or adenosine and ECS efficacy should be treated carefully unless they are peer-reviewed and independently replicated. Purinergic signaling is biologically relevant to seizure threshold, adenosinergic inhibition and neuroplasticity, but the clinical evidence that microbiota-derived adenosine determines ECT response remains insufficient [86,87]. Such work is useful for hypothesis generation and metabolomic panel design, not for clinical decision making.

## 7. Inflammation, Kynurenines and the Immune–Metabolic Bridge

The strongest current bridge between microbiome biology and ECT response is not a bacterial taxon. It is an immune-inflammatory phenotype. Higher baseline CRP and IL-6 have been associated with greater depressive symptom reduction after ECT in meta-analytic evidence [46,88]. This is a crucial observation because CRP and IL-6 are clinically accessible, mechanistically interpretable and biologically upstream or downstream of multiple microbiome-related processes [89,90].

Inflammation may influence ECT response through neuroplasticity, glial activation, neurovascular function, monoamine metabolism, HPA-axis tone and glutamatergic signaling [14,20,91]. ECT itself can induce acute immune and endocrine responses and may produce longer-term changes in inflammatory and neurotrophic systems [92]. The correct interpretation is not that inflammation is uniformly bad or that ECT simply reduces inflammation. A more nuanced model is that a subgroup of depression with elevated inflammatory tone may be especially sensitive to ECT-induced resetting of neuroimmune and neuroplastic networks [20,89].

Kynurenine biology is particularly important because it links inflammation, microbial metabolism and neural excitability. Pro-inflammatory signaling can shunt tryptophan metabolism toward kynurenine pathway metabolites [72,93]. Quinolinic acid may influence glutamatergic neurotoxicity, whereas kynurenic acid can modulate NMDA receptor signaling. ECT studies have reported changes in kynurenine pathway metabolites in treatment-resistant depression, and some data suggest that kynurenine metabolite dynamics may relate to symptom change [32,94,95]. Microbes can influence tryptophan availability, indole production and immune regulation, making kynurenines a biologically coherent component of a microbiome-informed ECT panel [93,96].

From a clinical standpoint, kynurenines have a better future as part of a panel than as a single biomarker. A patient with high CRP, high IL-6, altered KYN/TRP ratio, low butyrate-producing capacity, constipation and short EEG seizures under benzodiazepine exposure represents a different biological context from a patient with low inflammatory markers, normal bowel transit, no anticonvulsant exposure and robust seizures [46,97]. Precision ECT should capture these patterns rather than seek one universal marker.

The same logic applies to IL-8, TNF-alpha and IL-1beta. These markers may be relevant, but current clinical ECT evidence is less mature than for CRP and IL-6 [46,98]. Future studies should use prespecified immune panels, fasting blood draws where feasible, batch correction, symptom timing relative to treatment, and repeated measures that can distinguish baseline prediction from acute postictal immune response [20,72].

## 8. Short-Chain Fatty Acids, Gut Barrier Biology and Functional Microbiome Readouts

Functional readouts are central to microbiome-informed ECT research because microbial metabolites are more clinically interpretable than genus-level taxonomic patterns alone [99,100]. Short-chain fatty acids (SCFAs), particularly butyrate, acetate and propionate, are central candidates because they influence epithelial barrier function, immune tone, microglial maturation, energy metabolism, HPA-axis signaling and neuroplasticity [97,101,102].

Butyrate is especially relevant because of its role in intestinal barrier integrity and histone deacetylase inhibition [103,104]. In theory, reduced butyrate-producing capacity could promote low-grade inflammation, gut permeability and altered immune signaling, creating a biological context in which ECT response and cognitive tolerability differ. However, this remains a hypothesis in ECT. It should be tested with measured fecal and plasma metabolites, not inferred from the presence or absence of a few butyrate-associated taxa [11,97].

Gut barrier biology is another plausible but methodologically difficult pathway. Lipopolysaccharide-related signaling, lipopolysaccharide-binding protein, soluble CD14, intestinal fatty-acid binding protein and zonulin-like markers are often invoked as indicators of microbial translocation or barrier dysfunction [9,10]. These markers can support a gut–immune model, but they are not interchangeable and some, particularly commercial zonulin assays, require cautious interpretation [105,106]. In ECT cohorts, barrier markers should be analyzed alongside constipation, gut transit, medications, diet and systemic inflammation [107].

Indoles and bile acids are also relevant. Microbial indole derivatives can influence epithelial barrier function, aryl hydrocarbon receptor signaling and immune regulation [108,109]. Bile acid metabolism links gut microbiota to metabolic syndrome, inflammation and central signaling [110,111,112]. In patients receiving ECT, these pathways may be confounded by obesity, diabetes, metformin, diet, antipsychotic exposure and hospitalization. This is precisely why metabolomics should accompany metagenomics in future studies. Microbial tryptophan catabolites and SCFA-related signaling provide particularly plausible functional readouts for this type of integrated model [99,113].

The methodological implication is clear. Shotgun metagenomics is preferable to 16S rRNA sequencing when the research question concerns functional capacity, strain-level variation or microbial metabolic pathways [53,114]. If 16S is used, it should be treated as a screening tool, not a definitive functional assay. Relative abundance data must also be handled as compositional data, with appropriate statistical methods and clear reporting of normalization, batch correction and multiple-comparison control [115,116,117].

## 9. HPA Axis, Autonomic Regulation and the Vagus Nerve

The HPA axis offers a second bridge between microbiome biology and ECT physiology. Foundational microbiome work showed that microbial colonization can shape stress-reactivity systems in early life [118,119,120]. Subsequent gut–brain research has linked microbial states with stress responsiveness, autonomic regulation and immune–endocrine signaling [99,121]. ECT, in turn, produces acute autonomic and endocrine responses, including hypothalamic and cardiovascular activation [122]. These systems converge on the same clinical variables: seizure threshold, postictal recovery, delirium vulnerability, appetite, sleep, gut motility and treatment tolerability [123,124,125].

In severe depression, HPA-axis dysregulation is common but heterogeneous. Some patients show hypercortisolemia, sleep–wake disruption, weight loss and psychomotor slowing; others have metabolic syndrome, chronic inflammation and atypical features [123,126]. The microbiome may shape this endocrine context through microbial metabolites, vagal afferents, immune signaling and barrier function [99,121,127,128]. However, HPA biomarkers are rarely integrated into ECT–microbiome studies. This is a missed opportunity.

The vagus nerve is especially relevant because it connects gut physiology with central autonomic and affective circuits [129]. Dai and colleagues’ ECS–vagotomy findings provide a concrete translational signal that seizure-based neuromodulation can affect colonic motility through vagal pathways [81]. In future human studies, heart-rate variability, bowel motility, constipation measures and autonomic medication burden could help connect microbial findings to ECT physiology [122,130,131]. This would be more informative than taxonomy alone.

Clinically, autonomic state is visible. A patient with severe psychotic depression may present with dehydration, tachycardia, poor intake, constipation and insomnia. A catatonic patient may have immobility, reduced bowel sounds, and a high risk of medical complications. A clozapine-treated patient may have profound gastrointestinal hypomotility [132,133]. These states alter both the conduct of ECT and the validity of stool microbiome data [134]. Future studies should not sanitize them away; they should measure them [135,136].

## 10. Neuroplasticity, Brain Networks and the Biological Context of Therapeutic Seizures

ECT’s therapeutic effect is commonly conceptualized through neuroplasticity. Human neuroimaging studies consistently show structural and functional brain changes during ECT, including hippocampal and amygdala volume changes, network-level alterations and associations between brain changes and clinical outcome [15,45,76]. These findings do not reduce ECT to a single mechanism; rather, they indicate that induced seizures produce broad plasticity-related effects across mood and cognitive circuits [1,137].

Microbiome-informed ECT adds a contextual hypothesis: microbial, immune and metabolic states may influence the brain’s capacity to translate seizure-induced perturbation into adaptive plasticity. Inflammation can prime microglia, alter neurotrophin signaling, affect glutamatergic balance and modify cortical–striatal or limbic network function [12,14,91]. SCFA can influence microglial maturation and immune tone [138,139]. Kynurenine metabolites can affect NMDA receptor-related signaling [31,140]. HPA-axis state can influence neurogenesis, sleep and cognitive recovery [1,123,141]. These pathways are individually plausible; their ECT-specific integration remains untested.

A useful clinical analogy is wound healing. The same procedural stimulus can produce different outcomes depending on the host inflammatory, metabolic and endocrine state. ECT-induced seizure activity may similarly occur within a biological host context that shapes the durability and direction of neuroplastic response [12,92]. This analogy should not be pushed too far, but it clarifies why microbiome markers should be considered as contextual variables rather than causal replacements for established ECT mechanisms.

Future studies should therefore combine microbial and immune–metabolic measures with ECT-relevant brain outcomes where feasible: baseline cognition, postictal reorientation, autobiographical memory, mood-network imaging, hippocampal change, resting-state connectivity or electrophysiological metrics [15,45,137]. The field will not advance if microbiome data are collected in isolation from the neurobiology that ECT already measures.

## 11. Seizure Biology, ECT Parameters and Anesthetic Confounding

Any microbiome-informed ECT model that ignores seizure biology will fail clinically. The induced seizure is the proximate physiological event. Its quality is shaped by seizure threshold, stimulus dose, pulse width, electrode placement, anesthetic agent, medication exposure, ventilation, physiological state and number of treatments [4,142]. The microbiome may eventually prove relevant to this physiology, but only if studies record ECT parameters with sufficient precision [1,42,44].

The registry study by Gillving and colleagues is particularly useful because it places seizure duration back into the prediction conversation at scale [42]. The study found that EEG seizure duration was associated with remission in major depressive disorder and that anticonvulsant and benzodiazepine exposure was associated with shorter seizures and less favorable outcomes [42,142]. The implication for microbiome studies is direct: if non-responders have shorter seizures because of benzodiazepines, anticonvulsants or anesthetic depth, a microbial non-responder signal may be spurious unless these variables are modeled [143,144,145].

Anesthetic choice is not a technical afterthought. Propofol, methohexital, thiopental, etomidate, ketamine and esketamine differ in anticonvulsant properties, hemodynamic profile, recovery characteristics and potential neurobiological effects [146,147,148]. Propofol may shorten seizures; etomidate may prolong them but can have recovery and side-effect trade-offs; ketamine and esketamine have independent antidepressant and glutamatergic effects [142,148,149]. The anesthetic–ECT time interval and depth of anesthesia can also influence seizure dynamics [4]. In microbiome-informed research, anesthetic agent and dose should be recorded at the session level [4,150].

Ketamine deserves special attention because it intersects with depression, ECT, inflammation and cognition. Ketamine-assisted ECT has been evaluated in systematic reviews and network meta-analyses, but benefits are not uniformly established and cognitive effects require caution [148,149,151,152,153]. If a future ECT–microbiome study includes ketamine or esketamine anesthesia, it must prespecify whether ketamine is considered an anesthetic exposure, antidepressant co-intervention or mechanistic confounder. Otherwise, microbial or inflammatory signals could be incorrectly attributed to ECT [148].

Medication effects remain relevant to seizure physiology. Benzodiazepines and anticonvulsants may reduce seizure adequacy or shorten seizure duration [42,143,144,145]. Lithium may influence delirium risk or cognitive tolerability [154,155]. Antipsychotic and anticholinergic exposure may affect gastrointestinal physiology, constipation risk, metabolic status and microbial ecology [62,156,157,158]. These exposures are discussed in greater detail in the pharmacomicrobiomics section.

## 12. Cognitive Tolerability and the Microbiome: An Underdeveloped Endpoint

Response and remission are necessary but insufficient outcomes for precision ECT. Cognitive tolerability is central to patient-centered decision making. ECT can produce transient cognitive impairment, acute postictal disorientation and, in some patients, autobiographical memory disturbance [1,159,160]. Risk is influenced by electrode placement, pulse width, stimulus dose, treatment frequency, baseline cognition, age, concomitant medication and illness severity [1,160]. A precision model that predicts symptom response but ignores cognitive tolerability is incomplete [161,162,163].

Microbiome-informed ECT has not yet adequately addressed cognition. This is a major gap. Microbial and inflammatory states may plausibly influence cognitive vulnerability through systemic inflammation, HPA-axis dysregulation, sleep disruption, delirium susceptibility, metabolic syndrome, gut barrier signaling and neurovascular function [164,165,166,167]. These pathways are speculative in ECT but biologically coherent. They justify measurement, not clinical claims.

The practical endpoint should not be a vague statement that cognition improved or worsened. Studies should include orientation time, delirium or prolonged confusion, MoCA or comparable global cognitive measure, verbal memory where feasible, and a structured measure of autobiographical memory [159,168]. Baseline cognitive function should be recorded before treatment because it may predict both ECT response and vulnerability to adverse cognitive effects [169,170,171].

From a clinician’s perspective, the relevant question is not whether the microbiome causes memory problems. It is whether a combined phenotype—older age, high inflammatory markers, metabolic syndrome, constipation, sedating medications, lithium exposure, poor sleep, low baseline cognition and bilateral electrode placement—predicts a higher risk of cognitive adverse effects [172,173]. Microbial data may eventually add to this risk model, but they must be tested against variables already known to matter [172,174,175].

## 13. Psycho-Pharmacomicrobiomics and Medication Exposure

Pharmacomicrobiomics is central to this review because ECT is rarely delivered in medication-free patients. In severe depression and psychotic disorders, patients often receive antidepressants, antipsychotics, mood stabilizers, benzodiazepines, anticonvulsants, anticholinergic agents, proton-pump inhibitors, laxatives and sometimes antibiotics. Each can affect either the microbiome, ECT physiology or both [62,143].

A systematic review and meta-analysis of psycho-pharmacomicrobiomics concluded that psychotropic medications are associated with altered gut microbiome composition and that the gut microbiome may, in turn, influence psychotropic efficacy and tolerability [62]. This is highly relevant to ECT cohorts because medication exposure may differ between responders and non-responders for reasons unrelated to microbial biology. For example, a patient with severe anxiety and insomnia may receive higher benzodiazepine doses, leading to shorter seizures and lower remission probability, while also differing in diet, sleep and gut physiology [145,176].

Antipsychotics are particularly important. Olanzapine has been linked in animal and clinical literature to microbiome changes and metabolic dysfunction; clozapine is strongly associated with constipation and gastrointestinal hypomotility [64,67,158]. In a clozapine-treated patient referred for ECT, stool microbiota may reflect slow transit and laxative exposure as much as psychiatric pathophysiology [177,178]. Reporting clozapine dose without documenting bowel habit is inadequate for microbiome interpretation [131].

Antidepressants and mood stabilizers also matter. SSRIs, SNRIs, tricyclics, lithium and valproate may influence gut function, inflammation, appetite, weight or seizure threshold [179,180,181]. Proton-pump inhibitors can alter oral and gut ecology [182]. Antibiotics can cause large and prolonged microbial shifts [176,183]. Metformin, often used in antipsychotic-associated metabolic syndrome, has well-described microbiome effects [183]. Future ECT–microbiome studies must therefore treat medication exposure as a biological dataset, not merely as a demographic descriptor [85,184].

Microbiome signals in ECT studies cannot be interpreted without medication stratification. At minimum, investigators should record current and recent antibiotics, PPIs, probiotics, laxatives, benzodiazepines, anticonvulsants, lithium, antipsychotics, antidepressants, anticholinergics, metformin and anesthetic agents, with dose, duration and timing relative to sampling and treatment [53,176].

## 14. Gastrointestinal Phenotype: Constipation, Transit Time and Hospital Ecology

Gastrointestinal phenotype is not peripheral to microbiome-informed ECT. It is one of the determinants of microbiome validity. Constipation, slow transit, dehydration, reduced mobility, low food intake, anticholinergic burden, opioid exposure, clozapine treatment and hospital diet can substantially alter stool microbiota and microbial metabolites [185,186,187]. In severe psychiatric illness, these factors are common rather than exceptional [64,67].

The Dai study provides a rare ECT-specific gastrointestinal signal [81]. The clinical observation of improved gastrointestinal symptoms, particularly constipation, and the ECS–vagotomy component in mice suggest that ECT may modulate gut motility via autonomic pathways [81]. If so, stool microbiome changes after ECT may partially reflect altered transit time. This is not a problem; it is a biological observation. But it means that transit must be measured [82].

Future studies should include a minimum gastrointestinal panel: bowel movement frequency, Bristol Stool Scale, Rome-style constipation criteria where feasible, laxative exposure, anticholinergic burden, diet and fiber intake, clozapine/olanzapine exposure, physical activity or mobility status, inpatient versus outpatient setting, and timing of stool sampling relative to bowel movement and ECT session [132,135,178,188,189]. Without these data, stool microbiome findings are uninterpretable.

Hospital ecology should also be acknowledged as a care-setting confounder rather than as an ECT-specific microbiome mechanism [33,135,189]. Inpatient ECT may standardize medication administration and meal timing, but it may also introduce changes in mobility, sleep, institutional diet, infection exposure, bowel regimen, hospitalization duration before sampling, and acute medication exposure [85,135,176,182,189]. Each of these variables can influence stool consistency, gut transit, microbial composition, or microbial metabolite profiles [82,83,84,135,185,186,187]. Outpatient ECT has a different confounding structure, including more variable diet, oral-health routines, home bowel regimen, medication adherence, physical activity, and timing of sampling relative to meals, bowel movement, and ECT sessions [54,55,135,189]. Therefore, inpatient/outpatient status and hospitalization duration before sampling should be recorded and modeled together with medication exposure, diet, stool form, gut transit, constipation measures, laxative use, and oral-health metadata [33,54,55,82,83,84,85,134,135,176,182,189].

## 15. Mechanistic Synthesis: From Microbial Ecology to Seizure-Induced Neuroplasticity

A coherent model requires two directional pathways. Pathway A is microbiome-to-ECT response. In this model, baseline oral and gut microbial ecology influence microbial metabolites, gut barrier function, immune activation, HPA-axis tone, vagal afferent signaling, kynurenine metabolism, glutamate/GABA balance, microglial priming, BDNF-related plasticity and cortical–limbic network responsiveness [11,97,99]. These factors may then shape seizure threshold, EEG seizure duration, postictal suppression, cognitive tolerability and clinical response [41,82,83].

**Figure 1:**
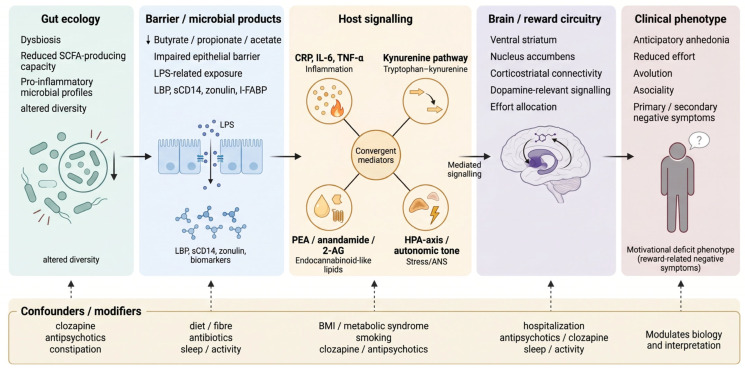
Microbiome-informed precision ECT model. The figure summarizes two directional pathways relevant to microbiome-informed ECT research: baseline oral and gut microbial ecology as a potential contextual predictor of ECT response and ECT-induced seizure physiology as a potential driver of secondary gastrointestinal and microbial changes. The model links microbial metabolites, gut barrier function, immune-inflammatory signaling, kynurenine biology, HPA/autonomic regulation, seizure physiology and clinical outcomes, while emphasizing major confounders including ECT parameters, anesthesia, medication exposure, diet, oral health and gastrointestinal phenotype. The clinical-phenotype endpoint panel is intended as an illustrative transdiagnostic endpoint space across severe depression, psychotic depression, catatonia and schizophrenia-spectrum presentations, rather than as a diagnosis-specific outcome model. In the trial framework proposed in this review, severe unipolar or bipolar depression remains the primary target population, with schizophrenia/catatonia considered exploratory cohorts. Created in BioRender. Szarpak, L. (2026). https://BioRender.com/xreuwxy (accessed on 22 June 2026).

Pathway B is ECT-to-gut physiology. The induced seizure triggers autonomic, hypothalamic, endocrine and neuroimmune responses [14]. These may alter appetite, gut motility, constipation, vagal signaling and inflammatory tone [20,81]. Subsequent microbial changes may therefore be secondary to changes in host physiology rather than primary mechanisms of psychiatric improvement. This distinction is essential because it determines study design. A conceptual framework linking baseline oral and gut microbial ecology with immune–metabolic signaling, gastrointestinal physiology, ECT-induced seizure biology and clinical outcomes is shown in Figure 1.

Session-level ECT variables should be captured in detail: electrode placement, pulse width, stimulus charge, seizure threshold, EEG seizure duration, postictal suppression, anesthetic agent and dose, number of sessions, treatment frequency and continuation or maintenance ECT [41]. Medication and clinical confounders should be prespecified: diet, BMI, smoking, alcohol use, antibiotics, probiotics, proton-pump inhibitors, laxatives, benzodiazepines, anticonvulsants, lithium, antipsychotics, clozapine/olanzapine exposure, anticholinergic burden, periodontal disease, oral hygiene and constipation. The minimum confounder domains and reporting requirements for microbiome-informed ECT research are summarized in Table 3.

Applied to this study design, Figure 1 should be read as a temporal and covariate map rather than as a causal claim. The microbiome-to-ECT pathway requires baseline oral and stool sampling before the first treatment, prespecified clinical and tolerability endpoints, blood inflammatory and metabolomic measures, and session-level ECT variables. The ECT-to-gut pathway requires post-treatment sampling aligned with ECT sessions, bowel movement timing, appetite, diet, mobility, laxative exposure, anesthesia, medication changes and gastrointestinal phenotype. In this framework, every microbiome measurement should be linked to sampling compartment, timing, clinical outcome, seizure quality, medication/anesthesia exposure and gastrointestinal context.

**Figure 2:**
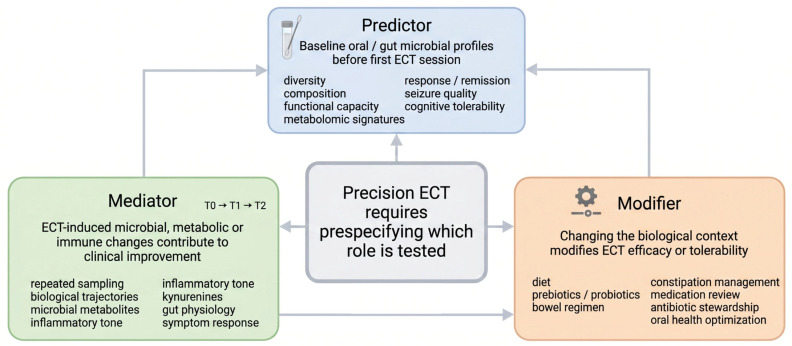
Predictor–mediator–modifier framework for microbiome-informed ECT research. The figure distinguishes three non-equivalent roles of microbiome findings in ECT studies: baseline microbial profiles as candidate predictors, treatment-associated microbial or metabolic changes as candidate mediators, and externally modified microbial or gastrointestinal context as a candidate modifier of ECT efficacy or tolerability. These roles require different study designs and should not be interpreted interchangeably. Created in BioRender. Szarpak, L. (2026). https://BioRender.com/a7mw7kn (accessed on 22 June 2026).

A predictor hypothesis asks whether baseline microbiome or metabolomic features identify patients more likely to respond to or tolerate ECT. This requires sampling before the first treatment, prespecified responder definitions and adjustment for clinical predictors, CRP/IL-6, medication and ECT parameters [22,190]. A mediator hypothesis asks whether ECT-induced microbial or metabolic changes contribute causally to improvement. This requires repeated measures, temporal modeling, mediation analysis and ideally experimental support [191]. A modifier hypothesis asks whether changing the microbial context—through diet, bowel regimen, prebiotics, probiotics, antibiotic stewardship or medication review—improves efficacy or tolerability. This requires interventional trials [192]. The distinction between predictor, mediator and modifier hypotheses is illustrated in Figure 2.

Figure 2 provides a practical classification rule for future studies. If oral or gut microbiome measures are obtained before ECT and tested against response, remission, seizure quality or cognitive tolerability, the study addresses a predictor hypothesis. If serial microbial, metabolic or immune changes are tested as mechanisms linking ECT exposure with clinical improvement, the study addresses a mediator hypothesis and requires repeated sampling, temporal precedence and mediation analysis. If diet, bowel regimen, oral health, probiotics, prebiotics, antibiotic stewardship or medication review are manipulated to improve ECT efficacy or tolerability, the study addresses a modifier hypothesis and requires a controlled interventional design. These roles should be prespecified before microbiome associations are interpreted.

Most existing evidence is compatible with the predictor hypothesis but insufficient for mediation or modification claims. The oral microbiome pilot and schizophrenia stool study both suggest baseline differences without robust pre–post shifts [24,63]. The ECS studies support gut inflammatory and motility mechanisms [11,81]. The inflammatory meta-analysis indicates that CRP and IL-6 are more mature candidates than microbiome measures [46,89]. Together, these findings argue for multimodal prediction rather than microbiome determinism.

The most plausible future biomarker will probably not be a single bacterium. It will be a composite: clinical phenotype, CRP/IL-6, kynurenines, SCFAs or metabolomic signatures, oral and stool microbiome features, constipation/transit data, medication exposures and seizure-quality metrics [41,50,192,193]. The incremental value of each layer must be tested explicitly.

## 16. Toward a Microbiome-Informed Precision ECT Trial

The next step should not be another very small pre–post microbiome study. The decisive question is incremental prediction: does adding oral/gut microbiome and metabolomic information improve prediction beyond clinical phenotype, inflammatory markers and ECT seizure parameters [41,49]? If it does not, microbiome testing will remain biologically interesting but clinically redundant. A minimum design framework for a future microbiome-informed precision ECT cohort is proposed in Table 4.

A credible prospective study would enroll 150–250 patients with severe unipolar or bipolar depression referred for ECT, with an exploratory schizophrenia or catatonia cohort [8,48]. Severe depression should be the primary population because outcome definitions are better standardized, ECT indications are common and comparison with existing predictors is feasible [7,41]. Psychosis/catatonia cohorts should be phenotyped separately rather than collapsed into a single severe-mental-disorder category.

**Figure 3:**
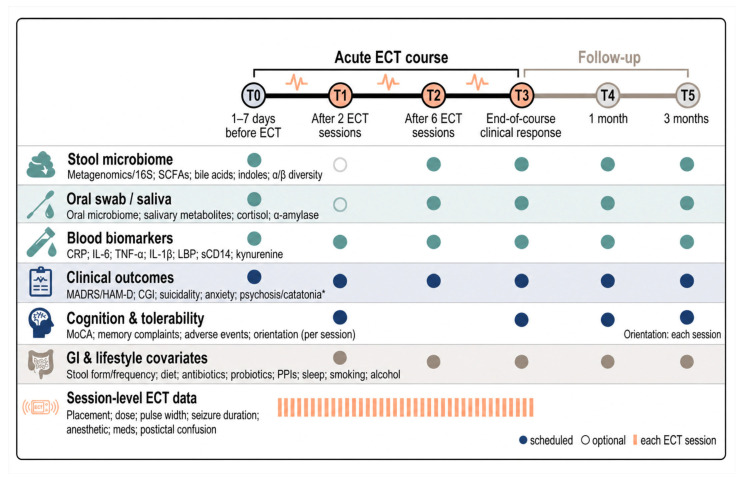
Sampling timeline for a future microbiome-informed ECT cohort. The figure illustrates a longitudinal assessment framework with baseline sampling at T0 (1–7 days before the first ECT treatment), followed by repeated evaluations after two sessions (T1), after six sessions (T2), at the end of the acute course (T3), at 1 month (T4), and at 3 months (T5). Stool, oral/salivary samples, blood biomarkers, clinical scales, cognitive measures, gastrointestinal phenotype and session-level ECT parameters are aligned temporally across the study course. The design is intended to distinguish baseline predictors from early biological change, end-of-course response and later relapse or tolerability signals. Created in BioRender. Szarpak, L. (2026). https://BioRender.com/5z6g3qi (accessed on 22 June 2026).

Sampling should include stool, oral swab or saliva and blood at baseline, after two ECT sessions, after six sessions, at the end of the acute course, at one month and at three months [47,49]. Baseline sampling is essential for prediction. Early sampling can detect biological change before clinical remission [194]. End-of-course sampling can distinguish treatment response from exposure effects. Follow-up sampling can address relapse, maintenance treatment and persistent cognitive or gastrointestinal outcomes [1]. A proposed longitudinal sampling and assessment schedule for a microbiome-informed precision ECT cohort is shown in Figure 3.

The primary endpoint should be response, defined as at least a 50% reduction in MADRS or Hamilton Depression Rating Scale score, and remission using accepted cutoffs [40,195]. Secondary endpoints should include speed of response, number of sessions to remission, relapse after ECT, cognitive tolerability, autobiographical memory, postictal reorientation, gastrointestinal symptoms, constipation and adverse events [1,160]. This outcome set reflects actual clinical decisions: not simply will the patient improve? but how quickly, how completely, with what cognitive cost, and with what maintenance plan?

Microbiome analysis should preferably use shotgun metagenomics, with 16S rRNA sequencing as a minimum if resources are constrained [196]. Oral and gut compartments should be analyzed separately. Metabolomics should include SCFAs, bile acids, indoles, kynurenines and purines/adenosine-related metabolites [47,197]. Immune measures should include CRP, IL-6, TNF-alpha, IL-1beta, LBP and soluble CD14 [20,194]. Gut barrier markers such as zonulin and intestinal fatty-acid binding protein may be included but must be interpreted cautiously.

For future primary ECT–microbiome studies, reporting should include STORMS-aligned microbiome metadata rather than only taxonomic results. At minimum, investigators should report the sampling compartment, collection device, time from collection to freezing, storage temperature, extraction kit, sequencing platform, negative and positive controls, contamination-control strategy, bioinformatic pipeline, normalization approach, batch handling, and missing-data handling. Clinical metadata should include recent antibiotics, probiotics/prebiotics, proton-pump inhibitors, laxatives, bowel frequency, Bristol Stool Scale, diet or fiber estimate, oral-health status, hospitalization duration before sampling, and session-level ECT/anesthesia variables [33].

Session-level ECT variables should be captured in detail: electrode placement, pulse width, stimulus charge, seizure threshold, EEG seizure duration, postictal suppression, anesthetic agent and dose, number of sessions, treatment frequency and continuation or maintenance ECT [41,136]. Medication and clinical confounders should be prespecified: diet, BMI, smoking, alcohol use, antibiotics, probiotics, proton-pump inhibitors, laxatives, benzodiazepines, anticonvulsants, lithium, antipsychotics, clozapine/olanzapine exposure, anticholinergic burden, periodontal disease, oral hygiene and constipation [198]. The minimum confounder domains and reporting requirements for microbiome-informed ECT research are summarized in Table 5.

Statistical modeling should compare clinical-only prediction with clinical plus inflammatory biomarkers and then with clinical plus inflammatory plus microbiome/metabolome plus ECT seizure-parameter models [199,200]. Nested cross-validation, calibration assessment, overfitting control and external validation should be used where possible [201]. Decision-curve analysis would help determine whether microbial testing changes clinical decisions enough to justify cost and complexity.

## 17. Clinical Implications

The immediate clinical implication is restraint. Microbiome testing should not be used to decide whether a patient is referred for ECT, which electrode placement is chosen, how stimulus dosing is set or whether continuation of ECT is prescribed [41,202]. There is no validated oral or stool microbial biomarker for ECT decision making [41,46]. Given the very small size of the direct human ECT–microbiome literature, the term “microbiome-informed precision ECT” should be understood as a research framework rather than a clinically deployable strategy; current data support hypothesis generation and baseline risk stratification research but not microbiome-guided ECT decision making.

A second implication is that routine ECT research assessments should become more microbiome-aware even before microbial testing enters clinical practice. Clinicians already document diagnosis, medications, anesthesia and seizure duration [1,41]. Future research protocols should similarly document constipation, laxative use, PPI exposure, recent antibiotics, smoking, hospital diet, oral health, clozapine/olanzapine exposure and anticholinergic burden [18]. These variables improve interpretation even if no microbiome assay is performed.

A third implication is that inflammatory markers are the most realistic near-term bridge. CRP and IL-6 are inexpensive, clinically accessible and supported by more direct ECT outcome evidence than microbial taxa [46,89,90]. Microbiome-informed ECT should therefore be developed as a multimodal prediction framework, not as a replacement for clinical assessment or inflammatory phenotyping [41,203].

Finally, the clinician’s language matters. Patients should not be told that their microbiome predicts ECT response. A scientifically accurate formulation is that researchers are studying whether gut and oral microbial patterns, inflammation and gastrointestinal physiology may one day help personalize ECT [202]. This distinction protects patients from overclaiming and protects the field from premature translation.

## 18. Clinical Readiness and Limitations

### 18.1. Why This Is Not Clinically Actionable Yet

Microbiome-informed precision ECT is not currently ready for clinical decision making. The direct human evidence is limited to very small studies, including a 14-patient oral microbiome pilot, an 11-patient schizophrenia stool microbiome cohort, and a single case report. These observations have not been independently replicated, have not been validated in adequately powered prospective cohorts, and do not establish microbiome mediation of ECT response. No study has yet shown that oral or gut microbiome measures improve prediction beyond established clinical variables, inflammatory markers, medication exposure, anesthesia, and seizure-quality parameters. Similarly, there are no prospective modifier trials showing that changing diet, bowel regimen, oral health, probiotics, prebiotics, or microbial ecology improves ECT efficacy or tolerability. For these reasons, microbiome findings should currently be interpreted only as hypothesis-generating candidate contextual markers, not as tools for ECT referral, protocol selection, or treatment modification.

### 18.2. Methodological Limitations of the Current Evidence

Beyond the lack of current clinical actionability, the evidence base remains methodologically immature. Available studies are small, diagnostically heterogeneous and difficult to compare because of varied ECT protocols, different sampling compartments, inconsistent microbiome methods, limited functional resolution and incomplete confounder control. Baseline microbial differences may reflect age, diet, smoking, medication exposure, oral health, constipation, hospitalization or inflammatory comorbidity, while post-treatment microbial changes may reflect altered appetite, mobility, bowel transit, hospital diet or medication changes rather than ECT-specific biology [18,29]. The current translational readiness of candidate microbiome-related markers and multimodal precision-ECT models is summarized in Table 6.

The oral microbiome depression pilot and the gut microbiome–schizophrenia cohort are important because they open the field, not because they settle it [24,63]. Neither provides a clinical biomarker. The Clostridium case report is a clinical observation, not causal evidence. ECS animal studies support plausibility but cannot establish human mechanisms or treatment recommendations [11].

Causality is unresolved. Baseline microbial differences may reflect age, diet, smoking, medication, oral health, constipation, hospitalization, inflammatory comorbidity or illness severity [18,29,53]. Pre–post microbial changes may reflect altered appetite, mobility, bowel transit, hospital diet or medication changes rather than ECT-specific biology. Taxonomic changes should not be overinterpreted without metabolomics, immune markers and clinical context [53,204,205].

There is also a risk of publication bias. Positive pilot findings are more likely to appear than null studies, especially in a young field [18,53]. Future studies should report negative results, share analytic pipelines when possible and avoid taxon-by-taxon storytelling unsupported by correction for multiple testing or functional validation [29,205].

Another limitation is that microbiome studies can create an illusion of precision. Sequencing produces thousands of features, but high-dimensional data in small samples are vulnerable to overfitting [18,53]. A microbial classifier built on 14 or 11 patients is not a biomarker; it is a hypothesis. Top-tier research will require adequate sample size, independent validation, prespecified analysis and integration with clinical variables already known to influence ECT outcome [41,49].

## 19. Future Directions

Future research should move from descriptive microbiome comparisons to precision-ECT modeling. The key question is not whether ECT changes bacteria; it is whether microbial, immune and metabolic phenotypes improve prediction or mechanistic understanding of ECT response beyond standard clinical and seizure-related variables [41,203].

The highest priorities are replication of baseline oral alpha diversity findings in larger depression cohorts; stool and oral sampling in the same patients; integration of CRP, IL-6, kynurenines, SCFAs and gut barrier markers; rigorous control of medications and constipation; standardized seizure-quality metrics; cognitive follow-up; and external validation of prediction models [41,46,49]. Studies should also test whether bowel-regimen optimization, dietary stabilization or medication review improves interpretability before microbial interventions are attempted. Recent mechanism reviews, national outcome data and network/prediction analyses reinforce the need for integrative ECT models that combine biology with clinical and session-level data rather than relying on single-marker stratification [20,206].

Interventional microbiome modulation—probiotics, prebiotics, dietary fiber, synbiotics or targeted metabolic strategies—should not be combined with ECT as an efficacy-enhancing intervention until observational groundwork is stronger [207]. If tested, such interventions should be adjunctive, controlled, mechanistically measured and designed with attention to safety, timing, seizure threshold, antibiotic exposure and psychiatric acuity [208,209].

The field should also consider the oral microbiome as a practical clinical signal. Oral sampling may be easier to implement than stool sampling in ECT services [210]. However, its interpretation requires dental and periodontal metadata [210]. A future oral–microbiome classifier without oral-health measures would be clinically weak regardless of statistical significance [16,211].

Finally, future reports should make the predictor–mediator–modifier distinction explicit. A baseline-predictor study, a mechanistic mediator study and a microbiome-modification trial are different scientific projects [202,212]. Confusing them leads to overinterpretation. Separating them will make the field more credible [203,213].

## 20. Conclusions

Microbiome-informed precision ECT is biologically credible and clinically interesting, but it is not clinically actionable. Direct human evidence is limited to small pilot and early prospective studies, while the strongest current biological bridge comes from inflammatory markers, particularly baseline CRP and IL-6. Preclinical ECS studies support gut inflammatory, motility and vagal mechanisms, but they cannot substitute for human validation.

The most defensible conclusion is that oral and gut microbiome measures should be evaluated as part of the biological context in which ECT-induced seizure activity produces neuroplastic and clinical change. The future is unlikely to be a standalone microbiome test. A more realistic and clinically useful approach is multimodal stratification integrating clinical phenotype, inflammatory biomarkers, microbial and metabolic profiles, gastrointestinal phenotype, medication exposure and session-level ECT seizure parameters. The central task for the field is therefore not clinical microbiome-guided ECT, but rigorous testing of whether microbial and metabolomic data add incremental value to multimodal prediction models anchored in clinical phenotype, inflammation and seizure physiology.

For clinicians and researchers, the conceptual shift is simple but important: the microbiome should not be framed as an alternative mechanism of ECT, nor as a fashionable adjunct to a mature procedure. It should be studied as a possible contextual layer in precision ECT—one that may help explain why the same therapeutic seizure produces rapid remission in one patient, partial response in another and unacceptable tolerability in a third.

## Figures and Tables

**Table 1:** Search framework and evidence classification used for the structured narrative review.

Evidence Domain	Search Focus	Interpretive Role in this Review	Main Methodological Safeguards
Direct clinical ECT–microbiome evidence	Studies evaluating ECT in relation to oral, salivary, or stool microbiome measures; baseline versus post-treatment sampling; responder versus non-responder comparisons; associations with remission, cognitive outcomes, or gastrointestinal symptoms	Serves as the core empirical substrate for hypotheses positioning the microbiome as a candidate predictor of treatment response, a potential mediator of downstream biological effects, or a marker of post-ECT physiological change	Small and heterogeneous samples are interpreted conservatively; oral and gut compartments are analyzed separately; microbiome findings are not overinterpreted in the absence of rigorous control for medication exposure, diet, oral health, bowel habit, and ECT session parameters
Non-microbial ECT biomarker literature	Studies on inflammatory markers, kynurenine pathway metabolites, metabolomics, neurotrophins, endocrine markers, epigenetic measures, and blood-based predictors of ECT outcome	Provides the mechanistic bridge linking microbial biology to established biological correlates of ECT responsiveness, especially within immune-inflammatory-, metabolic-, and neuroplasticity-related pathways	These studies are not treated as microbiome evidence per se; they are incorporated only when a biologically plausible connection to microbial pathways can be articulated and when their relevance to ECT outcome is clinically meaningful
ECT seizure physiology and treatment-parameter evidence	Studies examining EEG seizure duration, seizure threshold, postictal suppression, electrode placement, pulse width, stimulus dosing, treatment frequency, concomitant psychotropics, and anesthetic agents	Defines the clinical and physiological backbone of any precision-ECT model and provides the contextual framework within which microbiome-related predictors must be interpreted	Session-level treatment variables are treated as essential covariates rather than optional descriptors; microbiome associations are considered uninterpretable if major seizure or treatment parameters are ignored
Gastrointestinal and autonomic phenotype evidence	Studies addressing constipation, bowel transit, gut motility, laxative exposure, gastrointestinal symptom burden, vagal signaling, and autonomic regulation in patients receiving ECT or related models	Functions both as a clinical outcome domain and as a major confounder domain, especially for interpretation of stool-based microbiome findings and for understanding gut–brain bidirectionality during ECT	Bowel habit, transit time, and bowel regimen are treated as core validity variables rather than ancillary clinical details; gastrointestinal features are explicitly integrated into microbiome interpretation whenever stool-based data are discussed
Preclinical ECS and translational gut–brain evidence	Animal studies using electroconvulsive shock (ECS) that assess gut microbiota, intestinal inflammation, microbial metabolites, gut motility, vagal mechanisms, neuroinflammation, or depressive-like behavior	Generates translational mechanistic hypotheses, especially concerning inflammation, autonomic signaling, intestinal physiology, and microbiome-related modulation of neuroplasticity	Preclinical findings are used for mechanism generation only and are not translated directly into clinical biomarker claims; species differences, model constraints, and limited bedside generalizability are made explicit
Broader microbiome-gut–brain and pharmacomicrobiomics literature	Literature on SCFAs, indoles, bile acids, tryptophan–kynurenine metabolism, HPA-axis regulation, microglial signaling, oral microbiome biology, intestinal permeability, and interactions between psychotropics/anesthetics and the microbiome	Supplies the biological plausibility framework and informs study design, interpretation of confounding, and prioritization of candidate pathways relevant to microbiome-informed precision ECT	Used selectively rather than encyclopedically; only evidence with direct relevance to ECT biology, psychiatric confounding, or candidate biomarker development is incorporated; mechanistic extrapolation is kept proportionate to the strength of available data

Note: The literature was organized into six predefined evidence domains to separate direct ECT–microbiome evidence from indirect biomarker, seizure-parameter, gastrointestinal, preclinical and broader gut–brain evidence. Abbreviations: ECS, electroconvulsive shock; ECT, electroconvulsive therapy; EEG, electroencephalographic; HPA, hypothalamic–pituitary–adrenal; SCFAs, short-chain fatty acids.

**Table 3:** Minimum confounder framework for microbiome-informed ECT research.

Level/Group	Domain	Variables	Minimum Reporting Requirement
Patient-level gastrointestinal phenotype	Gastrointestinal transit	Constipation, stool form, bowel frequency, laxatives, anticholinergic burden	Bristol Stool Scale, bowel frequency, constipation criteria and bowel regimen at each sampling point.
Patient-level oral phenotype	Oral ecology	Periodontal disease, dental status, oral hygiene, xerostomia	Dental/periodontal status, oral hygiene routine, xerostomia, recent dental procedures and timing of oral sampling.
Patient-level metabolic and lifestyle context	Diet–lifestyle–metabolic context	Diet, fiber intake, smoking, alcohol, BMI, diabetes, lipids, physical activity	Diet/fiber estimate, smoking status, alcohol use, BMI, metabolic comorbidity and physical activity or mobility status.
Microbiome-disrupting drugs	Direct microbiome modifiers	Antibiotics, proton-pump inhibitors, probiotics/prebiotics	Class, dose, indication, timing and washout; antibiotics documented for 8–12 weeks where possible.
Treatment-level medication exposure	Seizure-modifying medication	Benzodiazepines, anticonvulsants, lithium	Dose, timing before ECT, serum levels where relevant and continuation/withholding strategy.
Treatment-level medication exposure	Antipsychotic–metabolic exposure	Clozapine, olanzapine, other antipsychotics, metformin	Dose, duration, metabolic status, constipation and gut hypomotility management.
Treatment-level anesthesia exposure	Anesthesia	Propofol, methohexital, thiopental, etomidate, ketamine/esketamine	Agent, dose, anesthetic–stimulus interval and changes across sessions.
Treatment-level ECT procedure	ECT procedural variables	Electrode placement, pulse width, stimulus dose, seizure threshold, EEG duration, postictal suppression	Session-level reporting of technical parameters and seizure-quality variables.
Hospital and care-setting ecology	Inpatient/outpatient context	Hospitalization, institutional diet, sleep/activity changes, infection exposure, medication changes	Inpatient/outpatient status, hospitalization duration before sampling, major diet/activity changes and acute medication changes.

Notes: Bristol Stool Scale categories may be reported as type 1–2 indicating hard/lumpy stool and slow transit tendency, type 3–4 indicating formed stool, type 5 indicating soft blobs, and type 6–7 indicating loose or watery stool. Medication exposure should include dose, duration, indication, route, timing relative to microbiome sampling and ECT session, and recent initiation, discontinuation or dose change. Stool sampling should record timing relative to the last bowel movement, laxative use, antibiotics, meals and ECT session. Abbreviations: BMI, body mass index; ECT, electroconvulsive therapy; EEG, electroencephalographic.

**Table 4:** Minimum design framework for a future microbiome-informed precision ECT cohort.

Design Domain	Recommended Approach	Rationale	Minimum Reporting Standard
Cohort and clinical phenotyping	Enroll 150–250 patients with severe unipolar or bipolar depression referred for ECT; include an exploratory schizophrenia/catatonia cohort only if adequately characterized.	Provides sufficient scale for multivariable prediction while preserving a clinically coherent primary population.	Diagnosis, episode duration, severity, psychotic features, catatonia, treatment resistance, suicidality, prior ECT and inpatient/outpatient status.
Longitudinal sampling schedule	Collect stool, oral swab/saliva and blood at baseline, after two sessions, after six sessions, end of acute course, 1 month and 3 months.	Separates baseline prediction, early biological change, end-of-treatment response and relapse/maintenance signals.	Exact timing relative to ECT, anesthesia, meals, medications, bowel movement and antibiotic/laxative exposure.
Microbiome and metabolomic profiling	Analyze oral and stool compartments separately; use shotgun metagenomics where feasible, with 16S as a minimum; include SCFAs, bile acids, indoles, kynurenines and purine-related metabolites.	Moves the field beyond taxonomic description toward functional microbiome biology relevant to inflammation, seizure physiology and neuroplasticity.	Sampling protocol, storage conditions, sequencing platform, bioinformatic pipeline, contamination controls, batch effects and metabolomic assay methods.
Immune, gut barrier and autonomic measures	Measure CRP, IL-6, TNF-α, IL-1β, LBP, sCD14, selected gut barrier markers, cortisol/ACTH where feasible and HRV or other autonomic indices.	Captures the immune–metabolic bridge between microbial ecology, inflammatory depression, vagal signaling and ECT responsiveness.	Fasting status, sampling time, assay platform, missingness, inflammatory comorbidity and concurrent infection or anti-inflammatory treatment.
ECT procedure and seizure physiology	Record electrode placement, pulse width, stimulus charge, dosing relative to seizure threshold, EEG seizure duration, postictal suppression, anesthetic agent and number/frequency of sessions.	Distinguishes microbiome-related prediction from procedural and seizure-quality confounding.	Session-level ECT dataset, including anesthetic dose, benzodiazepines, anticonvulsants, lithium and major medication changes.
Clinical outcomes and tolerability	Assess response, remission, speed of response, cognitive tolerability, autobiographical memory, GI symptoms, constipation and relapse.	Precision ECT should predict not only antidepressant efficacy but also tolerability, GI phenotype and durability of benefit.	MADRS or HAM-D, remission definition, CGI, cognitive battery or MoCA, autobiographical memory measure, Bristol Stool Scale, bowel frequency and relapse criteria.
Prediction modeling and validation	Compare clinical-only models with clinical + inflammatory, clinical + inflammatory + microbiome/metabolome and full models including seizure-quality variables.	Tests whether microbiome data add clinically meaningful incremental value beyond established predictors.	Prespecified modeling plan, feature reduction, nested cross-validation, calibration, overfitting control, decision-curve analysis and external validation where possible.

Note: The microbiome-related reporting domains in this table are intended to be STORMS-aligned for future primary ECT–microbiome studies, particularly with respect to sampling metadata, laboratory workflow, sequencing and bioinformatic pipeline, contamination control, medication exposure, diet, gastrointestinal phenotype, oral-health metadata, and other clinically relevant confounders [32]. Abbreviations: ACTH, adrenocorticotropic hormone; CGI, Clinical Global Impression; CRP, C-reactive protein; ECT, electroconvulsive therapy; EEG, electroencephalographic; GI, gastrointestinal; HAM-D, Hamilton Depression Rating Scale; HRV, heart-rate variability; IL, interleukin; LBP, lipopolysaccharide-binding protein; MADRS, Montgomery–Åsberg Depression Rating Scale; MoCA, Montreal Cognitive Assessment; SCFAs, short-chain fatty acids; sCD14, soluble CD14; TNF-α, tumor necrosis factor-alpha; 16S, 16S ribosomal RNA gene sequencing.

**Table 5:** Mechanistic axes linking microbiome biology with ECT response and tolerability.

Mechanistic Axis	Candidate Readouts	Relevance to ECT Biology	Evidence Maturity	Implication for Future Studies
Immune-inflammatory tone	CRP, IL-6, TNF-α, IL-1β	May identify an inflammatory depressive phenotype more likely to benefit from ECT; interacts with sickness behavior, neuroplasticity and symptom reduction.	Moderate for CRP/IL-6 as ECT response predictors; microbiome-mediated causality remains unproven.	Include inflammatory markers as core covariates in all microbiome-informed ECT cohorts.
Tryptophan–kynurenine and microbial metabolite signaling	Tryptophan, kynurenine, KYNA, QUIN, KYN/TRP ratio, indoles, bile acids, purines	Links microbial metabolism, inflammation, glutamatergic signaling, neurotoxicity/neuroprotection and potentially seizure-related neurobiology.	Emerging human ECT metabolite evidence; microbiome link is biologically plausible but indirect.	Measure kynurenines and microbial metabolites alongside inflammatory markers rather than relying on taxa alone.
SCFA, gut barrier and endotoxin-related signaling	Butyrate, acetate, propionate, LPS, LBP, sCD14, zonulin, I-FABP	May connect dysbiosis, slow transit or reduced SCFA production with epithelial barrier dysfunction and systemic immune activation.	Strong general microbiome biology; direct ECT evidence remains limited.	Prioritize functional metabolomics and barrier markers but avoid overinterpreting isolated markers such as zonulin.
HPA–autonomic–vagal gut physiology	Cortisol, ACTH, HRV, bowel frequency, Bristol Stool Scale, gut motility measures	ECT induces autonomic and endocrine responses; vagal and motility pathways may mediate ECT-to-gut physiology and shape stool microbiome signals.	Foundational microbiome–HPA evidence and emerging ECT/ECS gut-motility data.	Assess GI phenotype, bowel transit and autonomic markers as both outcomes and confounders.
Neuroplasticity and brain network remodeling	BDNF, hippocampal/amygdala volume, synaptic markers, MRI indices	ECT-related clinical response is closely linked to neuroplastic mechanisms that may be modulated by inflammatory and microbial metabolic states.	Strong ECT neuroplasticity literature; microbiome contribution is indirect.	Model the microbiome as a biological context for ECT-induced plasticity, not as the sole therapeutic mechanism.
Seizure physiology and cognitive tolerability	Seizure threshold, EEG seizure duration, postictal suppression, cognitive scores, autobiographical memory	The therapeutic seizure is the primary physiological event in ECT; seizure quality, medications and systemic biology may influence response and cognitive adverse effects.	Strong clinical relevance for seizure variables; microbiome-specific evidence is currently absent.	Treat seizure-quality variables and cognition as mandatory endpoints in microbiome-informed prediction models.

Footnote: These mechanisms should not be read as parallel proof that the microbiome determines ECT outcome. They define biologically plausible axes through which microbial ecology, inflammation, gut physiology and seizure-related neuroplasticity may converge. The most robust future models will test these axes jointly rather than treating microbial taxa as standalone biomarkers. Abbreviations: ACTH, adrenocorticotropic hormone; BDNF, brain-derived neurotrophic factor; CRP, C-reactive protein; ECT, electroconvulsive therapy; EEG, electroencephalographic; GI, gastrointestinal; HPA, hypothalamic–pituitary–adrenal; HRV, heart-rate variability; I-FABP, intestinal fatty acid-binding protein; IL, interleukin; KYN/TRP, kynurenine-to-tryptophan ratio; KYNA, kynurenic acid; LBP, lipopolysaccharide-binding protein; LPS, lipopolysaccharide; QUIN, quinolinic acid; sCD14, soluble CD14; TNF-α, tumor necrosis factor-alpha.

**Table 6:** Translational readiness of candidate biomarkers for microbiome-informed precision ECT.

Candidate Marker/Model	Current Signal	Readiness Status	Recommended Use
Oral microbiome diversity	Pilot data suggest higher pre-treatment oral alpha diversity in ECT responders with severe or treatment-resistant depression.	Promising but preliminary. Not clinically actionable.	Replicate in larger cohorts with control for oral health, smoking, xerostomia, diet, medications and hospitalization.
Specific oral taxonomic profiles	Exploratory signals, including Streptococcus-dominant profiles in non-responders, have been reported in small pilot data.	Hypothesis-generating only.	Do not use clinically. Verify with species-level methods, standardized oral sampling and dental/periodontal assessment.
Gut microbial composition	A very small schizophrenia cohort linked baseline Bifidobacterium and Lactobacillus proportions with BPRS improvement after ECT.	Very early direct human evidence.	Replicate with shotgun metagenomics, absolute or functional profiling and rigorous control for antipsychotics, constipation, BMI, diet and hospitalization.
Inflammatory phenotype	Higher baseline CRP and IL-6 have stronger evidence as predictors of depressive symptom reduction after ECT.	More mature than microbiome markers but not sufficient as a standalone test.	Include CRP and IL-6 as core biological covariates in all microbiome-informed ECT studies.
Kynurenine and microbial metabolite pathways	ECT metabolite studies and microbiome–gut–brain biology support relevance of tryptophan–kynurenine metabolism, SCFAs and related microbial metabolites.	Mechanistically plausible; direct ECT–microbiome evidence remains limited.	Measure kynurenines, SCFAs, bile acids, indoles and purine-related metabolites alongside inflammation and microbiome profiles.
Gastrointestinal phenotype	Emerging clinical and translational data suggest ECT may interact with constipation, gut motility and vagal pathways.	Clinically accessible but undermeasured.	Add bowel frequency, Bristol Stool Scale, laxative exposure, constipation criteria and anticholinergic burden to ECT cohorts.
Standalone microbiome test for ECT referral	No validated evidence supports using oral or stool microbiome testing to decide ECT eligibility or protocol.	Not ready and not justified.	Avoid clinical use, commercial overclaiming and causal language.
Multimodal precision-ECT model	The strongest future framework combines clinical phenotype, CRP/IL-6, metabolomics, microbiome profiles, GI phenotype and seizure-quality variables.	Most realistic translational direction.	Test incremental predictive value over clinical and seizure-parameter models using cross-validation, calibration and external validation.

Note: No microbiome marker is currently ready for clinical decision making in ECT. The most defensible near-term strategy is not a standalone microbial biomarker but a multimodal model integrating microbial, inflammatory, metabolic, gastrointestinal and seizure-quality variables. Abbreviations: BPRS, Brief Psychiatric Rating Scale; CRP, C-reactive protein; ECT, electroconvulsive therapy; GI, gastrointestinal; IL, interleukin; SCFAs, short-chain fatty acids.

## Data Availability

No new data were created or analyzed in this study. Data sharing is not applicable to this article. The evidence discussed in this review is available in the published articles cited in the Reference list.

## Supplementary Materials

The following supporting information can be downloaded at: https://www.mdpi.com/article/10.3390/biomedicines14071467/s1, Supplementary Table S1: Key human and translational evidence informing microbiome-informed precision ECT.

## Abbreviations

The following abbreviations are used in this manuscript:

16S	16S ribosomal RNA gene sequencing
ACTH	Adrenocorticotropic hormone
BDNF	Brain-derived neurotrophic factor
BMI	Body mass index
BPRS	Brief Psychiatric Rating Scale
CGI	Clinical Global Impression
CRP	C-reactive protein
DBS	Deep brain stimulation
ECS	Electroconvulsive shock
ECT	Electroconvulsive therapy
EEG	Electroencephalography
GABA	Gamma-aminobutyric acid
GI	Gastrointestinal
HAM-D	Hamilton Depression Rating Scale
HPA	Hypothalamic–pituitary–adrenal
HRV	Heart-rate variability
I-FABP	Intestinal fatty acid-binding protein
IL	Interleukin
KYN/TRP	Kynurenine-to-tryptophan ratio
KYNA	Kynurenic acid
LBP	Lipopolysaccharide-binding protein
LPS	Lipopolysaccharide
MADRS	Montgomery–Åsberg Depression Rating Scale
MEDLINE	Medical Literature Analysis and Retrieval System Online
MoCA	Montreal Cognitive Assessment
MRI	Magnetic resonance imaging
NMDA	N-methyl-D-aspartate
PPI	Proton-pump inhibitor
PRISMA-ScR	Preferred Reporting Items for Systematic Reviews and Meta-Analyses extension for Scoping Reviews
QUIN	Quinolinic acid
rTMS	Repetitive transcranial magnetic stimulation
sCD14	Soluble CD14
SCFA	Short-chain fatty acid
SCFAs	Short-chain fatty acids
SNRI	Serotonin–norepinephrine reuptake inhibitor
SSRI	Selective serotonin reuptake inhibitor
STORMS	Strengthening The Organization and Reporting of Microbiome Studies
tDCS	Transcranial direct current stimulation
TNF-alpha	Tumor necrosis factor-alpha
VNS	Vagus nerve stimulation
