# Supplementary material for: Microbiome-Informed Precision Electroconvulsive Therapy: Oral–Gut–Immune Signatures and Seizure Biology as Candidate Predictors of Response—A Narrative Review
Source: Biomedicines. 2026 Jun 28;14(7):1467. doi: 10.3390/biomedicines14071467 (PMC13403508; doi:10.3390/biomedicines14071467)
Supplement: Supplementary file 1 [file biomedicines-14-01467-s001.zip › Supplementaty Table S1.zip!/Supplementaty Table S1.pdf]

Supplementaty Table S1. Key human and translational evidence informing microbiome-informed precision ECT

Evidence source	Design and biological material	ECT/outcome context	Main contribution to precision ECT	Evidentiary status
Ammer-Herrmenau et al., 2025 [1]	Prospective pilot cohort; severe or treatment-resistant depression; oral swabs before/after ECT; microbiome data in 14 patients.	Response defined as ≥50% MADRS reduction.	Higher pre-treatment oral alpha diversity in responders; no robust global post-ECT microbial shift. Supports a baseline oral-microbiome predictor hypothesis.	Direct human evidence, but pilot-level only; exploratory taxonomy; oral health, smoking, diet and medication confounding remain important.
Kanayama et al., 2024 [2]	Prospective cohort; 11 patients with schizophrenia; stool microbiota before/after ECT.	BPRS symptom improvement.	Higher baseline Bifidobacterium and lower Lactobacillus associated with greater improvement; no major pre-post microbiome change.	Direct human stool evidence, but very preliminary; limited confounder control and functional resolution.
Kanayama et al., 2019 [3]	Single case report; schizophrenia; stool microbiota before/after ECT.	Individual ECT course.	Decrease in Clostridium abundance after ECT.	Hypothesis-generating only; no causal or biomarker inference possible.
Dellink et al., 2025 [4]	Meta-analysis; inflammatory markers in ECT-treated depression; 14 studies, 556 patients; no microbiome sampling.	Depressive symptom reduction after ECT.	Higher baseline CRP and IL-6 associated with greater symptom reduction. Provides the strongest current immune-inflammatory bridge to microbiome-informed ECT.	Indirect microbiome evidence; supports multimodal models but not microbiome causality.
Gillving et al., 2024 [5]	Population-based registry; 6998 patients with MDD treated with unilateral ECT; no	EEG seizure duration, medications and remission.	EEG seizure duration associated with remission; anticonvulsants and benzodiazepines associated with	Indirect but essential; establishes seizure quality and medication exposure as

	microbiome sampling.		shorter seizures and poorer outcomes.	mandatory covariates.
Dai et al., 2025 [6]	Clinical MDD component plus depressive-like mouse model; GI symptoms, gut motility and vagal pathway assessment.	ECT/ECS in relation to constipation and motility.	ECT associated with improved GI symptoms; ECS effects on colonic motility involved subdiaphragmatic vagal pathways. Supports an ECT-to-gut physiology pathway.	Translational support; not a microbiome biomarker study; requires prospective validation.
Ji et al., 2025 [7]	CUMS depressive-like mouse model; gut microbiome and intestinal cytokines.	ECS/ECT-like treatment and depressive-like behavior.	ECS improved depressive-like behavior, reduced gut TNF-α, IL-6 and IL-1β, and altered microbial diversity/taxa.	Preclinical mechanism-generating evidence only; taxa cannot be directly translated to human ECT.

References:

1. Ammer-Herrmenau C, Hamm J, Neesse A, Gunther K, Besse M, Zilles-Wegner D. Response to electroconvulsive therapy is associated with a more diverse oral microbiome: a prospective longitudinal cohort pilot study. Eur Arch Psychiatry Clin Neurosci. 2025. doi:10.1007/s00406-025-01976-3.
2. Kanayama M, Nagahama M, Otsuki K, Miyaoka T, Horiguchi J, Inagaki M. The gut microbiota response to electroconvulsive therapy for schizophrenia: a prospective cohort study. Shimane J Med Sci. 2024;41:83-91. doi:10.51010/sjms.41.3_83.
3. Kanayama M, Hayashida M, Hashioka S, Miyaoka T, Inagaki M. Decreased Clostridium abundance after electroconvulsive therapy in the gut microbiota of a patient with schizophrenia. Case Rep Psychiatry. 2019;2019:4576842. doi:10.1155/2019/4576842.
4. Dellink A, Vanderhaegen G, Coppens V, et al. Inflammatory markers associated with electroconvulsive therapy response in patients with depression: a meta-analysis. Neurosci Biobehav Rev. 2025;170:106060. doi:10.1016/j.neubiorev.2025.106060.
5. Gillving C, Brus O, Larsson A, et al. Seizure duration and electroconvulsive therapy in major depressive disorder. JAMA Netw Open. 2024;7:e2422738. doi:10.1001/jamanetworkopen.2024.22738.
6. Dai M, et al. Electroconvulsive therapy improves distal colonic motility via the subdiaphragmatic vagus nerve in depressive-like mice. Sci Rep. 2025;15:19597. doi:10.1038/s41598-025-04114-0.
7. Ji J, et al. Mechanisms of microbial-gut-brain axis modulation by electroconvulsive therapy in the treatment of depression. Anesthesiol Perioper Sci. 2025. doi:10.1007/s44254-025-00103-8.
